# Advanced Age and Increased Risk for Severe Outcomes of Dengue Infection, Taiwan, 2014–2015

**DOI:** 10.3201/eid2908.230014

**Published:** 2023-08

**Authors:** Nicole Huang, Yi Jung Shen, Yiing Jenq Chou, Theodore F. Tsai, Chia En Lien

**Affiliations:** National Yang Ming Chiao Tung University, Taipei, Taiwan (N. Huang, Y.J. Chou);; Boston University, Boston, Massachusetts, USA (Y.J. Shen);; Office of the Deputy Superintendent, National Yang Ming Chiao Tung University Hospital, Yilan County, Taiwan (Y.J. Chou);; Takeda Vaccines, Cambridge, Massachusetts, USA (T.F. Tsai, C. En Lien)

**Keywords:** dengue, dengue risk factors, immunosenescence, flavivirus, viruses, Taiwan, *Suggested citation for this article*: Huang N, Shen YJ, Chou YJ, Tsai TF, Lien CE. Advanced age and increased risk for severe outcomes of dengue infection, Taiwan, 2014–2015. Emerg Infect Dis. 2023 Aug [*date cited*]. https://doi.org/10.3201/eid2908.230014

## Abstract

Dengue, a mosquitoborne flavivirus infection, is increasingly a disease of older adults who are more likely to have chronic diseases that confer risk for severe outcomes of dengue infection. In a population-based study in Taiwan, adjusted risks for dengue-related hospitalization, intensive care unit admission, and death increased progressively with age.

Dengue is an *Aedes aegypti* mosquito–borne flavivirus infection; a secular trend for dengue is a shift in the age of case-patients and deaths from children to adults and older adults (*1*,*2*). We report a population-based study of age-specific dengue risks for hospitalization, intensive care unit (ICU) admission, and death in Taiwan.

Dengue is not endemic in Taiwan but is introduced frequently; population immunity is low. In 2014–2015, large outbreaks led to 51,344 cases confirmed by the Taiwan Centers for Disease Control (Taiwan CDC) (*3*). In those years, suspected cases were tested at the Taiwan CDC by reverse transcription PCR, IgM/IgG, and paired IgM/IgG for seroconversion to confirm dengue infection; point-of-care NS1 antigen detection assays were introduced in September 2015. The database of reported cases was linked to the national administrative health database to report patient outcomes and to establish age-specific rates. Because reporting is required for reimbursement, the National Health Insurance Research Database provides near-complete ascertainment of all medical encounters for >99% of the island’s population. We adjusted for sex, socioeconomic status, and presence of underlying conditions (*2*). We did not review individual patients’ medical records in this linked-database analysis.

Age-specific attack rates during the outbreak were similar across age groups (Appendix Figure), consistent with low population immunity rates. Lower attack rates in children <10 years of age, in whom infections are more likely to be asymptomatic or mildly symptomatic, likely reflected a lower frequency of medical consultation and laboratory confirmation. We examined records of 51,344 case-patients, 15,847 of whom were hospitalized, in an analysis of healthcare utilization and deaths. Adjusted rates for hospitalization, ICU admission, and death within 30 days of confirmation of dengue illness increased sharply with advancing age, compared with rates among young adults (Figure). In contrast, case rates by age group were relatively flat.

**Figure:**
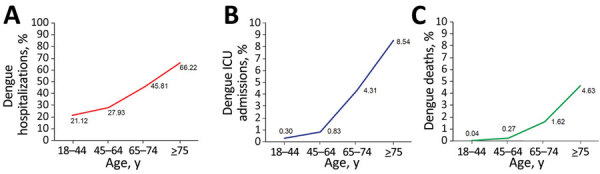
Dengue outcome event rates by age group in Taiwan. A) Hospitalizations; B) ICU admissions; C) deaths. ICU, intensive care unit.

The epidemiologic shift of dengue to adults and older adults has increased recognition that advanced age is a risk factor for severe and fatal outcome after dengue infection (*2*,*3*), adding dengue to the list of other flavivirus infections for which advanced age is well established to be a cardinal risk factor for severe disease. Age-specific curves by age for clinically severe dengue, seasonal influenza, and COVID-19 are similar; however, mortality rates and case-fatality ratios for dengue are considerably lower (*4*,*5*). Case-fatality ratios for dengue in Pan American Health Organization countries are <0.05%; in southern Brazil, mortality rates for dengue are 10-fold to 100-fold lower than for seasonal influenza (*4*–*6*).

Adults and older adults comprise an increasing proportion of dengue cases in many countries, even as those populations reach ages where the prevalence of chronic diseases increases (*7*). Because chronic diseases contribute to more severe dengue outcomes, the overall effects of dengue attributed to those underlying conditions are increasing in turn. In adults, underlying diseases, specifically diabetes, chronic renal disease, and heart disease, are associated with higher relative odds for progression to severe disease than secondary infection (*8*). However, in administrative database studies in Mexico, Brazil, and Colombia, comorbidities were shown to confer a higher risk for death in hospitalized patients across the age spectrum (*8**,**9*). Our understanding of pathophysiological mechanisms associated with increased dengue severity in patients with comorbidities and advanced age is limited, as it is for patients with influenza and COVID-19.

Since 1990, dengue cases and deaths have increased disproportionately among adults >50 years of age compared with children <15 years of age, in whom the number and proportion of cases and, especially, deaths, have declined (*1*). As of May 2023, Asia accounts for most global dengue cases; it is also the fastest-aging region, containing more than half of the world’s population >65 years of age. Projections suggest that 10% of the total global population by 2060 will be persons >65 years of age living in Asia (*10*). Ongoing demographic trends and the 2 risk factors, advanced age and chronic disease, for developing severe to fatal dengue underscore the need to improve protection from dengue for older adults and persons with chronic diseases in all regions in which dengue is endemic, particularly in Asia.

Appendix Additional information about increased risk for severe outcomes of dengue infection, Taiwan, 2014–2015.
