# Supplementary material for: Advanced Age and Increased Risk for Severe Outcomes of Dengue Infection, Taiwan, 2014–2015
Source: Emerg Infect Dis. 2023 Aug;29(8):1701–2. doi: 10.3201/eid2908.230014 (PMC10370833; doi:10.3201/eid2908.230014)
Supplement: Appendix — Additional information about increased risk for severe outcomes of dengue infection, Taiwan, 2014–2015. [file 23-0014-Techapp-s1.pdf]

Article DOI: http://doi.org/10.3201/eid2908.230014

EID cannot ensure accessibility for supplementary materials supplied by authors. Readers who have difficulty accessing supplementary content should contact the authors for assistance.

Advanced Age and Increased Risk for Severe Outcomes of Dengue Infection, Taiwan, 2014–2015

Appendix

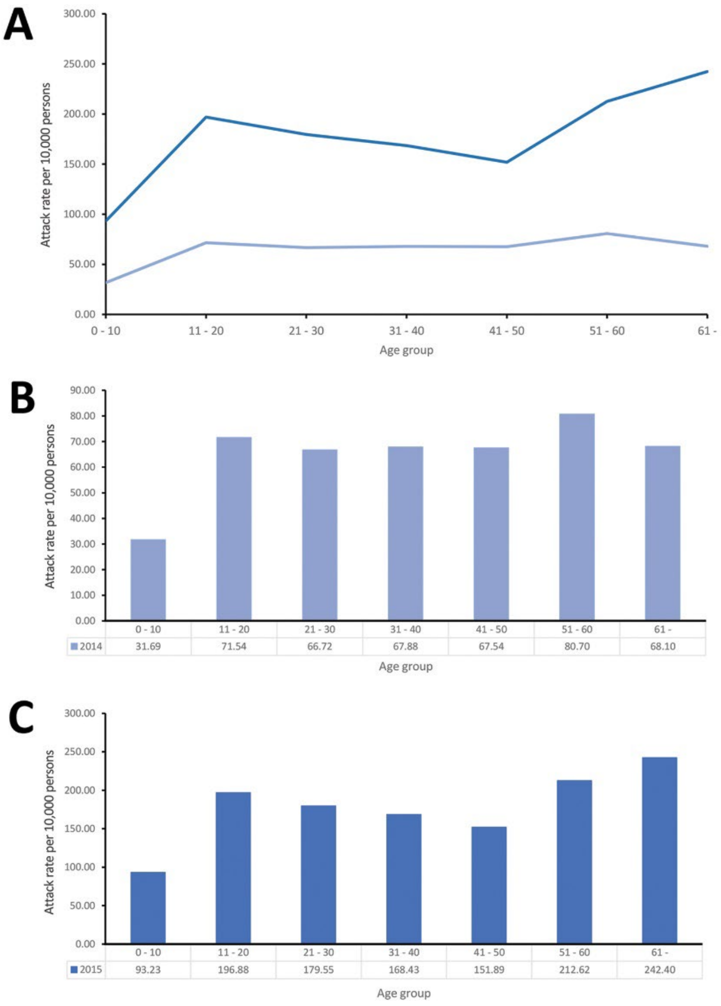

Appendix Figure. Age-specific attack rates for confirmed dengue cases in Taiwan during 2014 and 2015. The relatively uniform attack rates in all age groups are consistent with low population immunity. Source: https://www.cdc.gov.tw/File/Get/wSvkhxgGybm8szDWUtG3Jw.
